# Preparation of Ultrafine Fly Ash-Based Superhydrophobic Composite Coating and Its Application to Foam Concrete

**DOI:** 10.3390/polym12102187

**Published:** 2020-09-24

**Authors:** Huiping Song, Mingxiu Tang, Xu Lei, Zhengjun Feng, Fangqin Cheng

**Affiliations:** Institute of Resources and Environmental Engineering, Collaborative Innovation Center of High Value-added Utilization of Coal-related Wastes, Shanxi University, Taiyuan 030006, China; tangmx1997@163.com (M.T.); A1090821099@163.com (X.L.); fzj@sxu.edu.cn (Z.F.); cfangqin@sxu.edu.cn (F.C.)

**Keywords:** ultrafine fly ash (UFA), foam concrete, superhydrophobic, coating, waterproofing, 1H,1H,2H,2H-perfluorodecyltriethoxysilane (HFDS)

## Abstract

The waterproof and thermal insulation property of foamed concrete is very important. In this study, the ultrafine fly ash (UFA)-based superhydrophobic composite coating was applied onto foam concrete. The UFA-based base coating that closely adhered to the concrete initially improved the waterproofness of the test block, and the silane coupling agent-modified UFA-based surface coating further achieved superhydrophobicity. The UFA on the coating surface and the asperities on the surface jointly formed a lotus leaf-like rough micro–nanostructure. The 154.34° water drop contact angle and 2.41° sliding angle on No. 5 coating were reached, indicating that it was a superhydrophobic surface. The water absorption ratios of the composite coating block were 1.87% and 16.6% at 4 h and 7 days, which were reduced by 97% and 75% in comparison with the original foam concrete. The compressive strength and heat conductivity coefficient after soaking for 4 h of the composite coating block were higher than 4.0 MPa and 0.225 W·m^−1^·K^−1^, respectively. The UFA-based superhydrophobic composite coating proposed in this study and applied onto foam concrete is simple and cheap, requires no precise instrument, and can be applied in a large area.

## 1. Introduction

Lotus leaves and water skippers are well-known for their superhydrophobic surface [1,2]. The superhydrophobic surface has a contact angle greater than 150° and a sliding angle lower than 10° [3]. It has received extensive attention in the development of self-cleaning materials, and it has been studied in natural and artificial systems. These “lotus effect” structures possess waterproofing and self-cleaning [4], anticorrosion [5], antifog [6], and anti-ice properties [7]. Given these advantageous functions, the application of superhydrophobic materials in daily necessities, public buildings, national defense, and aviation have been extensively studied in applied research.

These exceptional functions are derived from the asperities of the surface micro-nanostructures and the wax-like substances on the surface [8]. In recent years, superhydrophobic surfaces have been developed rapidly, and superhydrophobicity can be achieved using various methods, such as template [9], etching [10], self-assembly [11], and sol–gel methods. The sol–gel method can be used to prepare superhydrophobic coatings in a large area with good heat resistance [12]. However, these coatings exhibit defects, such as easy to break and difficult-to-control thickness. Thus, popularizing the application of these coatings is difficult. Other methods require precise instruments, which translate to increased cost. Moreover, these methods are applicable to base materials, such as metal, glass, and fabrics, and cannot be produced and applied in building materials in a large scale. In practical application, the simplest surface modification method is the spray method, which does not require any special equipment or extreme conditions, such as high-temperature or high-vacuum condition. Furthermore, the spray method can achieve large-scale production, making it increasingly attractive.

With its light weight and heat preservation effects, the foam concrete is an ideal heat preservation material in buildings, thereby achieving the energy conservation of buildings. However, given its porous structure, the foam concrete has high water absorption rate and poor waterproofness. Once the water enters, the heat conductivity coefficient and heat preservation effect of the foam concrete increase and degrade, respectively [13,14]. In general, improving formulations or surface coatings could improve the stability and durability of concrete. Khosravani et al. [15] and Weinberg et al. [16] optimized the concrete composition through the performance test under dynamic load and improved the concrete performance and economic benefits. Liu et al. [17] treated concrete by adding powdery water repellents and coating liquid hydrophobic agent on the surface simultaneously in order to explore the water-resistant effect. However, the preparation method is relatively complex and expensive. A waterproof coating is usually sprayed on the external surface of the concrete to prevent the entry of water, and this strategy can evidently improve the heat preservation effect, corrosion resistance, and durability [18]. She et al. [19] have continuously stirred 100 g water, 76 g KOH, and 120 g phenyltriethoxy silane for 12 h at −20 °C to 0 °C and mixed the reaction product with 5 wt % SiO_2_ nanocolloid solution at 80 °C for 2 h to prepare the superhydrophobic emulsion for application onto the surface of cement concrete. However, a long-time operation under ultralow temperature is a tedious process with harsh conditions. Lu et al. [20] have reported the F-containing coupling reagent-treated TiO_2_ ethanol dispersion liquid coating. This coating, which has superhydrophobicity and superoleophobicity, can be applied onto the surfaces of all types of soft and hard materials. However, its application onto concrete surfaces has not been investigated thoroughly.

The purpose of this study was to explore a highly efficient and low cost waterproof coating applied onto a concrete surface, guaranteeing heat preservation and energy conservation properties. The superhydrophobic composite coating applied onto the foam concrete included two layers, ultrafine fly ash (UFA)-based superhydrophobic surface coating and waterproof base coating. Among them, the ultrafine fly ash (UFA) is used to construct a rough micro-nanostructure, and silane coupling reagent is used to reduce the surface energy. The composite coating could be large scale applied onto the surface of the foam concrete through the spray method exerting efficiently waterproofing and heat preservation effect with low cost.

## 2. Materials and Methods

### 2.1. Materials

The main materials of the waterproof base coating were styrene–acrylic emulsion (S400F, Basf Company), UFA (Shanxi Huatong Company, Tiaiyuan, China), and Portland cement (32.5R, Taiyuan Cement Company, Taiyuan, China). The functional additives included tributyl phosphate (defoamer, Wenhua Shanghai, Shanghai, China) and sodium hexametaphosphate (dispersant, Tianjin Chemical reagent, Tianjin, China).

The main materials of the superhydrophobic surface coating were absolute ethanol, 1H,1H,2H,2H-perfluorodecyltriethoxysilane (HFDS), 3-aminopropyltriethoxy silane (KH550), 3-(methylacryloxyl) propyltrimethoxy silane (KH570), phenyltriethoxysilane (PTES), and trimethylchloro silane (TMCS). All analytical reagents were purchased from the Guoyao Group (Shanghai, China). The bonding agent spray (3M) was purchased from Lekang (Shenzhen, China).

### 2.2. Experimental Methods

The superhydrophobic composite coating applied onto the foam concrete included two layers. The UFA-based waterproof and the superhydrophobic coatings served as base and surface coatings, respectively. The coatings were applied onto the surface of the foam concrete by using the spray method to exert waterproof effect. The UFA-based waterproof base coating was prepared as follows. Water (16 mL) was added to the styrene–acrylic emulsion (50 g). The mixture was mixed with 0.02 g dispersant and 0.02 g defoamer in a disperser (KS-370, Shanghai, China) for 1 min, added with 60 g UFA and 40 g cement, and stirred at 600 r min^−1^ for 5 min. After sitting for 2 min, the mixture was applied onto the surface of the foam concrete. The material was aged for seven days for the property test [5].

The superhydrophobic surface coating was prepared as follows. The silane coupling reagent (0–2 mL) was dissolved in ethanol (50 mL). The solution was added with UFA (0–10 g) and subjected to ultrasonic treatment by using an ultrasonic cleaner (KX-1730QT, Kexi, Beijing, China) for 20 min, and the superhydrophobic surface coating was obtained.

**Figure 1:**
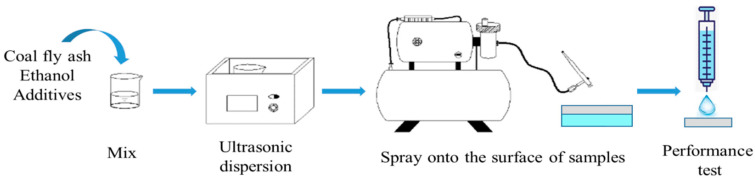
Preparation and application of the surface coating.

The superhydrophobic composite coating was applied as follows. The UFA-based waterproof base coating was sprayed/brushed onto the surface of the foam concrete test block, and the coating was approximately 2–3 mm thick. After drying at room temperature for one day, a layer of bonding agent spray and a layer of superhydrophobic surface coating were sprayed using an electric spraying machine (LJ-440S, Jinhua, Hangzhou, China). The use level was approximately 10 mL, and an analytical test was performed after drying at room temperature. The technological process is shown in Figure 1.

The chemical composition of UFA was measured using an X-ray fluorescence spectrometer (XRF, S8TIGER, Bruker, Karlsruhe, Germany) by using the direct compression method. The mean size analysis of the UFA particles was evaluated using a particle size analyzer (Eyetech/CIS, Ankersmid B.V., Samuel Morsestraat, Holland). The samples were characterized using Fourier transform infrared (FT-IR) spectroscopy (PerkinElmer Frontier, Waltham, MA, USA), tabletop scanning electron microscopy (SEM-EDS, TM3030Plus, Hitachi, Tokyo, Japan) and X-ray diractometer (XRD, D2 PHASER, Bruker, Karlsruhe, Germany), and atomic force microscopy (AFM, Nanosurf NANITE, Liestal, Switzerland).

**Figure 2:**
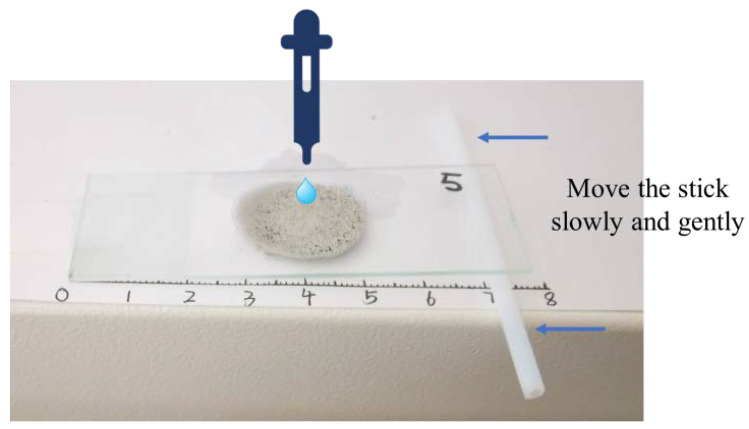
The test method for the sliding angle of the surface coating.

The coating samples of different formulas were prepared on glass slides by using a surface contact angle gauge (JC2000X6, Shanghai, China), and the water drop contact angle on the coating was determined using the sessile drop method. Water drops were discharged from the injector at 2–6 μL per drop. The equilibrium time utilized the essentially unchanged reading of contact angle as the criterion. The contact angle was measured at five points, and the average contact angle was obtained. The method of sliding angle detection is shown in Figure 2.

Slide coated with coating was placed on a flat surface marked from the left end of the slide to the right end. The left end of the slide was in contact with the paper surface and was fixed, and the right end was lined with a rolling cylinder with diameter *D*. A drop of water dripped over the coating, and the cylinder was gently pushed from right to left, stopping immediately when the water drop rolled. The drop was then marked *L* on the paper at the stop. At this point, the angle between the slide and the paper was the angle at which the droplets roll over the coating. The sliding angle of the droplet was calculated as Formula (1):

(1) $$\alpha =arctan\frac{D}{L}$$

where α is the sliding angle, °; *D* is the diameter of rolling cylinder, *m*; *L* is the distance between the rolling cylinder and the left end of the slide when the water drop rolled, *m*.

The flexibility, impact resistance, and bonding strength of the coating were tested in reference to the national standards “Determination of flexibility of films” (GB/T1731-93), “Determination of impact resistance of films” (GB/T1732-1993), and “Polymer-modified cement compounds for waterproofing membrane” (GB/T23445-2009), respectively.

**Figure 3:**
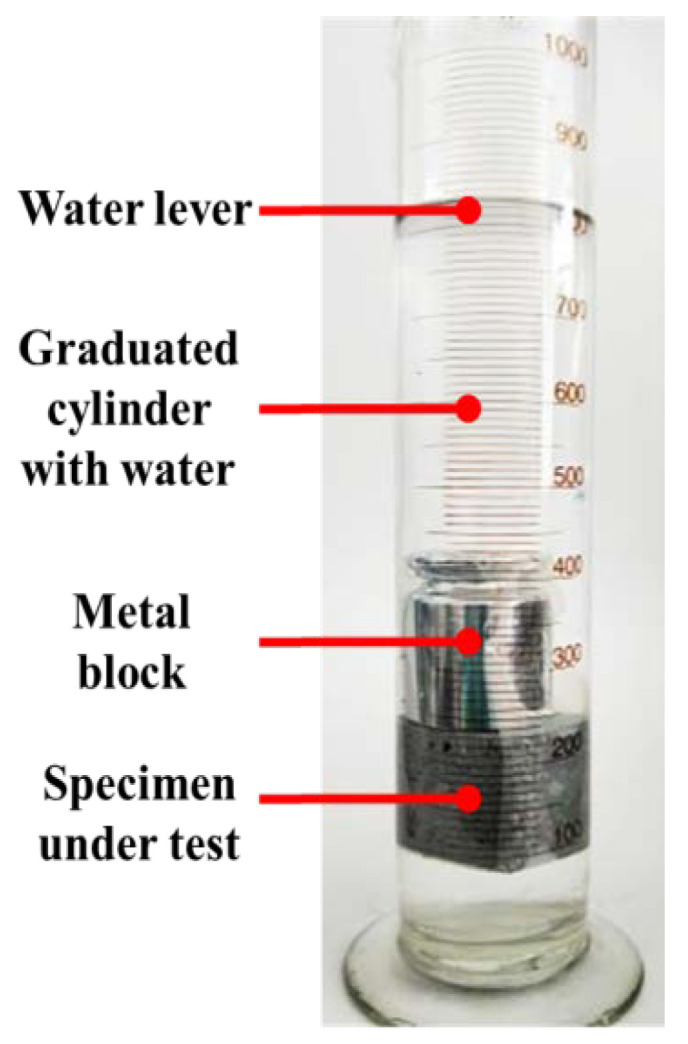
Determination of the water absorption of the foam concrete.

The foam concrete test block was placed in an air drying oven (GZX-GF-1-BS-II/H, Yuejin, Shanghai, China) and dried until a constant weight was achieved. The block was then collected and weighed (*m*_0_). The concrete test block was tied with a weight and rapidly placed into a measuring cylinder filled with a certain amount of water. The initial scale was recorded. The change in the liquid level in the measuring cylinder was observed within a certain time (Figure 3). The volume of liquid drawn was converted into the water mass absorbed by the test block, namely, the added mass of test block (*m*_1_) after water absorption. The average water absorption (*M_R_*) was calculated using Formula (2).

(2) $$M_{R}=\frac{m_{1}\;}{m_{0}}\times 100\%$$

Three dried test blocks (100 mm × 100 mm × 100 mm) were placed at the central position of the bottom plate of a compression testing machine (YAW-200B, Keweier Jinan, Jinan, China). The compressive direction of each test block was perpendicular to the expansion direction of the sample. The testing machine was started and loaded until the test block was fractured. The data were recorded, and the average compressive strength was determined. Dried standard test blocks (300 mm × 300 mm × 50 mm) were placed on an intelligent plate heat conductivity meter (DWR-1, Gangyuan, Tianjin, China). The testing machine was started, and the data were recorded. Three calculations were conducted in parallel, and the average heat conductivity coefficient was determined.

## 3. Results and Discussions

### 3.1. Analysis of UFA

**Figure 4:**
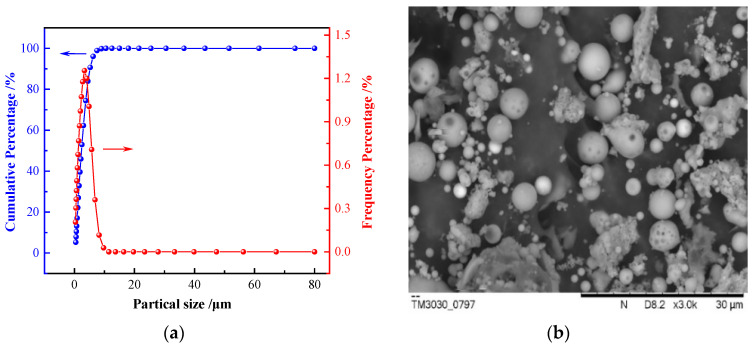

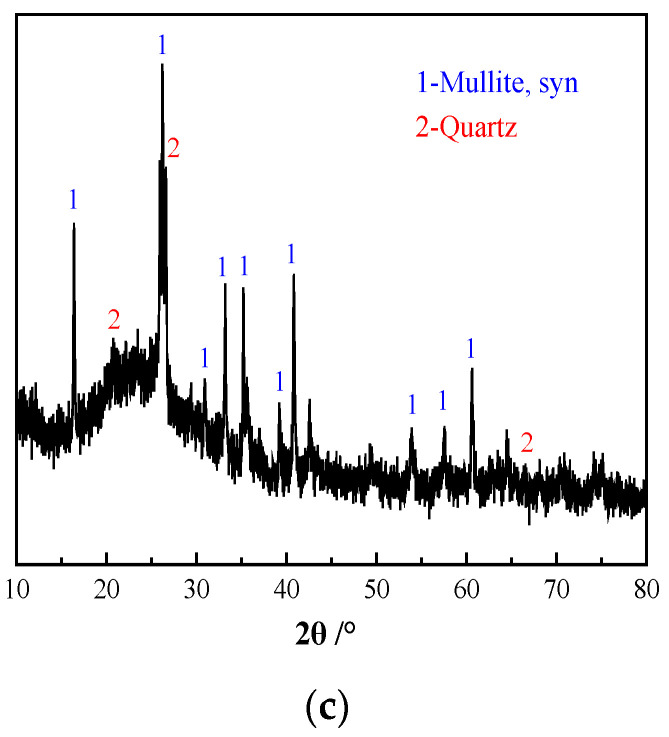
Analysis of ultrafine fly ash (UFA). (**a**) Particle size analysis; (**b**) SEM image; (**c**) XRD pattern.

The particle size analysis (Figure 4a) showed that the average size of UFA used in this study was approximately 2.35 µm. The particle size distribution was narrow, and over 90% of the particles were less than 5 µm. The SEM images showed that most UFA particles were spherical and smooth (Figure 4b), indicating good dispersibility. Table 1 lists the chemical composition of UFA determined using XRF. SiO_2_ and Al_2_O_3_ accounted for more than 84% of the total composition of UFA. The XRD analysis (Figure 4c) showed that the crystalline phases were mullite and quartz. Thus, coal fly ash has become a very important secondary resource, but attention should be paid to the negative effects on the environment and economy in the process of utilization [21]. Generally speaking, as long as the transportation radius is reasonable, fly ash is a good filler for concrete and coatings.

### 3.2. Hydrophobicity Analysis of the Superhydrophobic Coating

#### 3.2.1. Influence of the UFAs Modified Using Different Silane Coupling Reagents on the Coating Hydrophobicity

**Figure 5:**
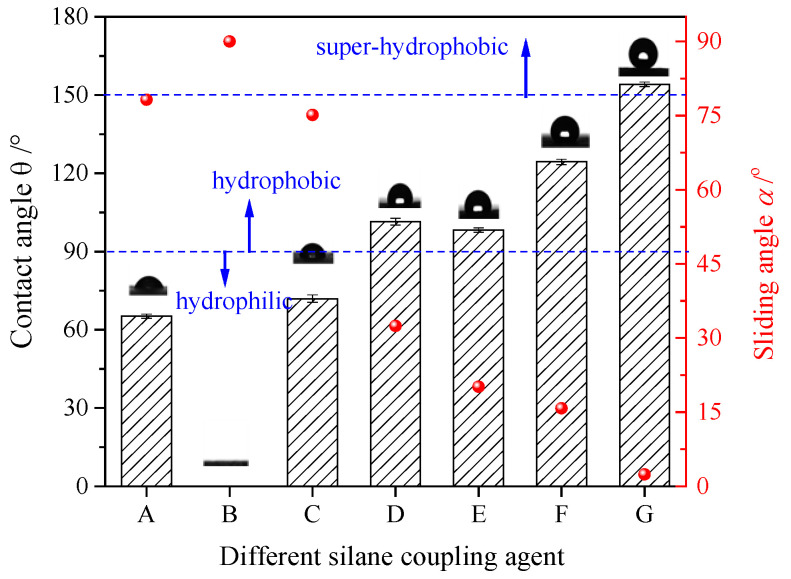
Influence of UFAs modified using different silane coupling reagents on the coating hydrophobicity. (Bar: contact angle, red dot: sliding angle; A-blank slide, B-UFA, C-PTES+UFA, D-KH550+UFA, E-KH570+UFA, F-TMCS+UFA, and G-HFDS+UFA; Preparation conditions: 1 mL silane coupling reagents, 5 g UFA, and 50 mL ethanol).

Different types of silane coupling reagents (1 mL) were dissolved in ethanol (50 mL), added with UFA (5 g), and subjected to ultrasonic treatment for 20 min. The reaction product was sprayed onto a glass slide presprayed with the bonding agent. The contact angle of the water drop was determined after drying. The hydrophobic effect is shown in Figure 5. The contact angle of the blank slide (A) surface was about 65.12°. The water drops on the coating surface obtained only by adding UFA in ethanol (B) were directly spread, and the contact angle was 0°. The contact angle of the coating added with PTES (C) was 71.93°, which was smaller than 90°, indicating that it was hydrophilic. The contact angle of the coating added with KH-550 (D), KH-570 (E), and TMCS (F) were 101.45°, 98.23°, 124.39°, respectively, indicating their conversion from hydrophilic into hydrophobic (90–150°). The contact angle of the coating added with HFDS (G) was 154.12°, and its sliding angle was less than 2.41°, indicating its superhydrophobicity due to high angle.

The contact angle of the HFDS coating was the largest, because, among the silane coupling reagents, HFDS had the lowest surface energy, extremely small F atom radius, low polarizability, and high electronegativity. The C–F bond had a large bond energy, small bond length, and low polarizability. The intermolecular force of the chemical compounds containing the C–F bond was small. As a result, the surface energy was small. All types of liquids had difficulty in wetting the coating, indicating good hydrophobic effect [22].

#### 3.2.2. Influence of the Amount of the HFDS Additive on the Coating Hydrophobicity and Analysis of the UFA Surface

**Figure 6:**
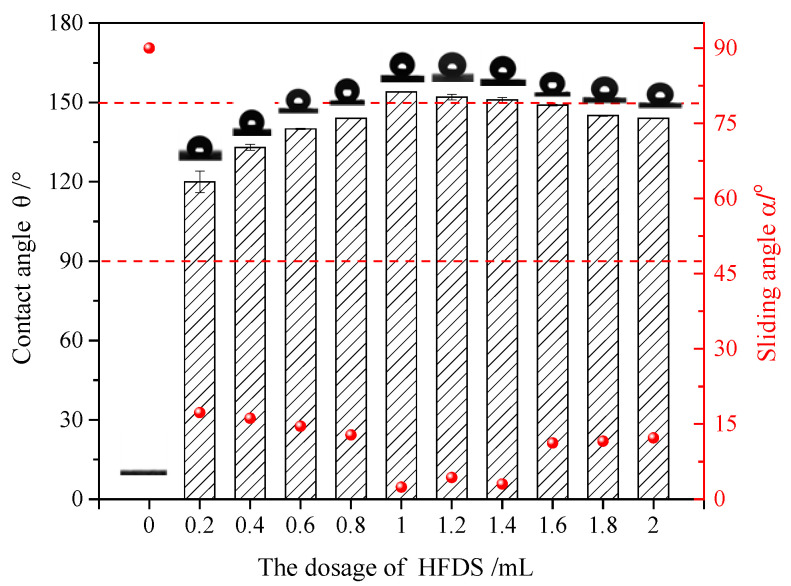
Influence of the amount of 1H,1H,2H,2H-perfluorodecyltriethoxysilane (HFDS) additive on the coating hydrophobicity (bar: contact angle, red dot: sliding angle; 5 g UFA, 50 mL ethanol).

The HFDS-modified UFA had the optimal superhydrophobic effect. Thus, the amount of the HFDS additive was investigated analytically. Varying amounts of HFDS were dissolved in 50 mL ethanol, and the solution was added with 5 g UFA. After 20 min of ultrasonic treatment, the reaction product was sprayed onto a glass slide presprayed with the bonding agent. The contact angle of the water drop was tested after drying. The hydrophobic effect is shown in Figure 6.

The water drops on the surface of the ethanol–UFA coating without HFDS were completely spread, producing a 0° contact angle. The contact angle of 0.2 mL HFDS coating increased to 120.32°, and the hydrophilic coating became hydrophobic. With increased amount of the HFDS additive, the coating hydrophobicity gradually improved. The contact angle of 1.0 mL HFDS coating reached 154.34°, and the sliding angle deduced to 2.4°, satisfying the required superhydrophobicity. This effect was due to the extremely low surface energy of HFDS, which reduced the surface energy of particles. Thus, the coating was difficult to dampen with water.

With increasing amount of the HFDS additive, the contact angle on the coating was reduced. This phenomenon was possibly because of the hydrolytic reaction of silicon hydroxyl (Si–OH) on the UFA surface, and the HFDS in the solution underwent hydrolysis and agglomeration to impede its hydrolysis with the UFA surface. Furthermore, with increased HFDS content, the probability of mutual collision among the HFDS molecules in solution was greater than the probability of collision between the HFDS molecules and the Si–OH on the UFA surface. Consequently, the grafting ratio of HFDS on the UFA surface was reduced, thereby influencing the hydrophobicity of the coating [23].

The addition of 1 mL HFDS into 50 mL ethanol with 5 g UFA was a good formula for surface coating. FT-IR spectroscopy and the SEM–EDS analyses were performed to verify the UFA modification by HFDS.

**Figure 7:**
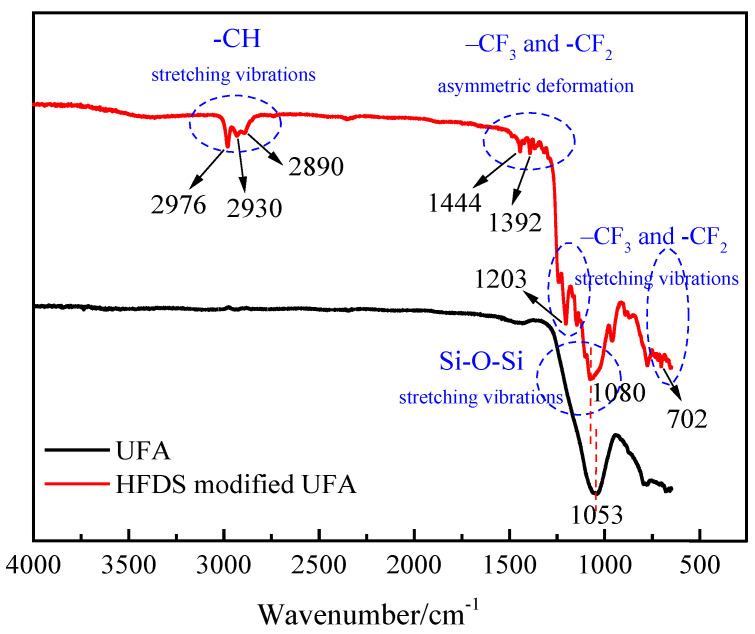
Structural configuration of UFA and HFDS modified UFA (1 mL HFDS, 5 g UFA, and 50 mL ethanol).

The FT-IR analysis of the groups on the UFA surface before and after HFDS-assisted modification was performed (Figure 7). The peak at 1053 cm^−1^ of the purified UFA sample corresponded to the internal SiO_4_ or AlO_4_ tetrahedra especially the vibrational stretching of Si–O and Al–O bonds in UFA [24]. The original peak moved toward a high wavenumber after modification, and a new absorption peak appeared at 1080–1100 cm^−1^. This new peak was caused by the Si–O–Si antisymmetric stretching vibration of the introduced silane coupling reagent. The new peaks at 702 and 1203 cm^−1^ were the –CF_3_ and –CF_2_ stretching vibrations [25]. The peaks at 1380, 1392, and 1444 cm^−1^ were caused by the asymmetric deformation of the –CF_3_ and the –CF_2_ groups [26]. The peaks at 2890, 2930, and 2976 cm^−1^ were due to the symmetric and antisymmetric stretching vibrations of –CH [27]. All these peak changes indicated that HFDS was grafted successfully onto the surface of the UFA particles.

**Figure 8:**
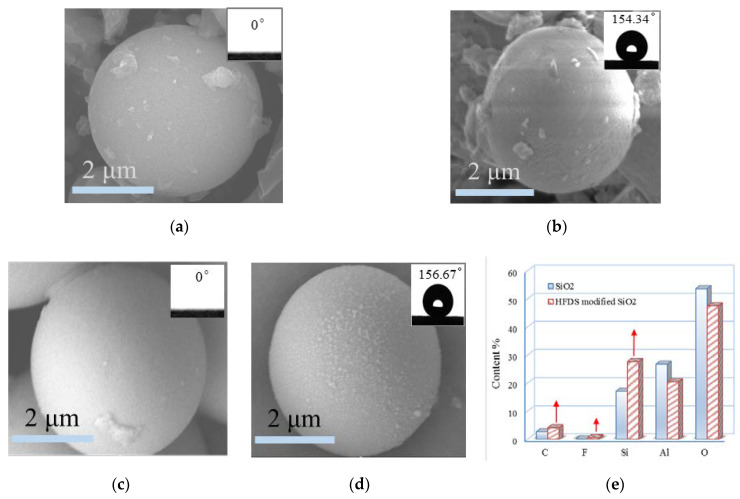
SEM–EDS comparison of particles (1 mL HFDS, 5 g UFA, and 50 mL ethanol). (**a**) UFA particle; (**b**) HFDS-modified UFA particle; (**c**) SiO_2_ particle; (**d**) HFDS-modified SiO_2_; (**e**) Comparison of EDS.

The surface microtopographies of the UFA particles before and after modification were analyzed via SEM (Figure 8a,b). In comparison with the unmodified UFA surface, tiny nanoasperities were found on the HFDS-modified UFA surface. However, given that the UFA surface was not that smooth, the asperities were not very evident or intuitive. SiO_2_ with similar composition, approximate particle size, and smooth surface (Figure 8c) was used as replacement of UFA in a comparison test to prove the structural change in the particle surface after HFDS-assisted modification, and many irregular asperities were observed clearly on the particle surface, as shown in Figure 8d. The SiO_2_ surfaces before and after HFDS-assisted modification were subjected to SEM–EDS. Figure 8e shows that the C, F, and Si contents on the modified surface increased due to the introduction of HFDS typical groups, such as –CF_2_, CF_3_, –Si–(O–). This finding agreed with the results of FT-IR spectroscopy. Therefore, HFDS provided the condition of low surface energy for the coating when grafted onto the particle surface.

#### 3.2.3. Influence of the UFA Dosage on the Coating Hydrophobicity and Hydrophobicity Analyses

**Figure 9:**
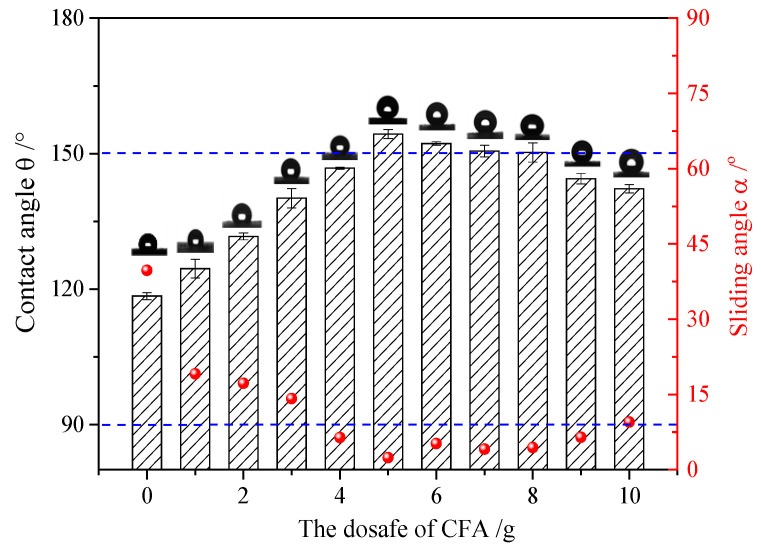
Influence of the UFA dosage on the coating hydrophobicity (bar: contact angle, red dot: sliding angle; 1 mL HFDS, 50 mL ethanol).

HFDS (1 mL) was dissolved in ethanol (50 mL), and the solution was added with different amounts of UFA. After 20 min of ultrasonic treatment, the reaction product was sprayed onto a glass slide presprayed with the bonding agent. The contact angle of water drop was tested after drying. The hydrophobic effect is shown in Figure 9. The samples were labeled as No. 1–10 specimen.

Without UFA, only the smooth HFDS coating was formed, and the water drop contact angle was 118.52°, indicating that the HFDS coating with extremely low surface energy showed hydrophobicity but not superhydrophobicity. With the addition of UFA (1–5 g), the water contact angle on the coating was gradually increased. The contact angle of the coating with 5 g UFA reached 154.34°, and its sliding angle was 2.41°, indicating that this coating is superhydrophobicity. Water droplet could roll off a glass slide coated with No. 5 coating and with a small angle, as shown in Video S1. When the amount of UFA was continuously increased above 5 g, the contact angle gradually decreased and the sliding angle increased.

The surface roughness values of the coatings are shown in Table 2. The No. 0 coating was basically similar to a smooth surface, and its surface mean square roughness (Sq) and water drop contact angle were only 0.3 nm and 118.52°, respectively. The surface of the No. 1 coating was uneven, and sparse particles were found. The Sq and contact angle of the No. 1 coating were 167 nm and 124.53°, respectively, which were higher compared with that of the No. 0 coating.

The rough structure on the surface of the No. 5 coating gradually became compact. The particles were aggregated, arranged on the surface, and interconnected to form polymers under random distribution. The nanoasperities covered the micron order upheaved structure. The Sq and water drop contact angle were 237 nm and 154.34°, respectively. These results may be because the micro-nanocomposite structure and the air gaps between particles existed on the coating surface to form the lotus leaf-like surface structure and achieve superhydrophobicity.

Large and small particles were lain together on the surface of the No. 10 coating and presented compact stacking state. Even with the presence of the rough micro-nanostructures on the surface, air gaps between the particles reduced, and Sq decreased to 215 nm. Contact angle of 142.22° were observed and the hydrophobic effect degraded. These results were due to the addition of UFA, and a certain amount of HFDS in the system was not enough to modify all particles. Given that UFA was hydrophilic, the unmodified UFA exposed on the coating surface degraded the hydrophobicity of the coating [28].

### 3.3. Theoretical Hydrophobicity Analysis of the Surface Coating

#### 3.3.1. Theoretical Analysis of the Surface Hydrophobicity of the Smooth HFDS Coating

The formation mechanism of self-assembled fluoroalkylsilane membrane has never been extensively investigated. It may be similar to the formation mechanism of the alkylsilane monolayer. In accordance with different reaction conditions, compact and orderly arranged alkyl chain momomolecular films can be formed or unself-assembled, and condensed and disorderly molecular coating can be formed [29].

**Figure 10:**
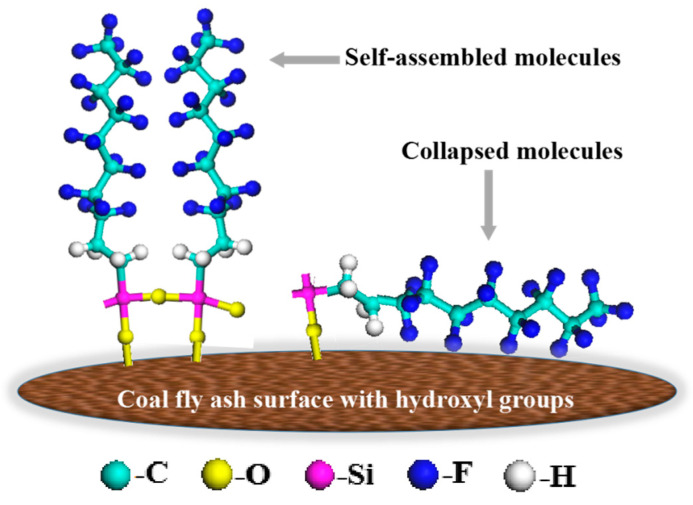
HFDS monolayer on the surface of coal fly ash with hydroxyl groups [29]. (Left: well-oriented or self-assembled, Right: collapsed molecules).

In this study, HFDS was dissolved in ethanol and underwent hydrolysis to generate products containing chemical groups, such as –CF_3_, –CF_2_, –CH_2_, and –Si–O–. Various arrangement modes may be available when HFDS-ethanol solution was sprayed onto the glass slide/mica sheet surface containing OH groups. The relative ratio of the ordered-phase arrangement to the disordered-phase arrangement depended on the HFDS concentration and the dispersive reaction time. Figure 10 displays the two possible arrangement modes of HFDS on the surface of coal fly ash. These modes are the monolayer disorderly collapsing molecular arrangement (Figure 10 right) and the orderly self-assembled structural domain (Figure 10 left). Furthermore, the –CF_3_, –CF_2_, and –CH_2_ groups exposed on the surface are displayed.

The wettability of the simplest chemically inhomogenous molecular-scale flat surface consisting of N phases can be calculated using Formula (3) [29].

(3) $${\left(1+\cos\theta \right)}^{2}=\overset{n}{\underset{i=1}{\sum }}f_{i}{\left(1+\cos\theta_{i}\right)}^{2}$$

where θ is the equilibrium contact angle on the chemically inhomogenous ideal smooth surface, θ*_i_* is the contact angle of one pure chemical homogenous phase occupying the area fraction of *f_i_*, and $\overset{n}{\underset{i=1}{\sum }}=1$.

The relationship between the self-assembly degree of HFDS on the surface of the base material and the water drop contact angle was analyzed using Formula (3) to speculate the arrangement mode of HFDS on the surface of base material. For the arrangement with low self-assembly degree (Figure 10 right), the groups exposed on the coating surface included one –CF_3_, seven –CF_2_, and two –CH_2_ groups with corresponding theoretical contact angles of water drops of 120°, 108°, and 94°, respectively [25]. Furthermore, the parameters were substituted into Formula (3).

$$(1+\cos\theta ){\;}^{2}=0.1\times {\left\{1+\cos\theta \left({\mathrm{CF}}_{3}\right)\right\}}^{2}+0.7\times {\left\{1+\cos\theta \left({\mathrm{CF}}_{2}\right)\right\}}^{2}+0.2\times {\left\{1+\cos\theta \left({\mathrm{CH}}_{2}\right)\right\}}^{2}\;$$

The theoretical contact angle under this arrangement mode was 105.7°.

For the orderly arrangement with high self-assembly degree (Figure 10, left), those exposed on the coating surface were the –CF_3_ groups, and the contact angle under ideal state was 120°.

The contact angle of the No. 0 coating was 118.52°, which was extremely close to the theoretical water drop contact angle of the –CF_3_ group (120°). HFDS may have an orderly arrangement when grafted onto the glass slide (Figure 10, left).

#### 3.3.2. Theoretical Hydrophobicity Analysis of No. 5 Coating

Any rough surface can be described using the fractal structure, which can achieve the quantitative characterization of the relationship between the coating surface microtopography and performance and the quantitative description of material surface microtopography [30,31]. Onda and Schibuichi [32] have investigated the relationship between fractal surface and wettability and proposed the wettability model (Formula (4)).

(4) $${\;\cos\theta }_{f}={\left(\frac{L}{l}\right)}^{D-2}\cos\theta_{s}$$

where $\theta_{f}$ is the theoretical contact angle of liquid drops on the fractal surface (HFDS-modified UFA coating surface), and $\theta_{s}\;$ is the intrinsic contact angle of the smooth HFDS surface. ${\left(\frac{L}{l}\right)}^{D-2}$ is the surface roughness factor, where *L* and *l* are the upper and the lower limits of the fractal dimension of the coating surface, respectively, and *D* is the fractal dimension of the coating surface.

The SEM graph of the No. 5 coating was analyzed using the box-counting method via the MATLAB software, and the *D* of the surface was acquired. The SEM graph was cropped into 512 × 512 pixels. The box size was changed in accordance with the amplification factor of the SEM graph and the series of corresponding values of the grid side length (*r*) and grid number (*N*[*r*]) covering the fractal particles, which conformed to the relationship indicated in Formula (5).

**Figure 11:**
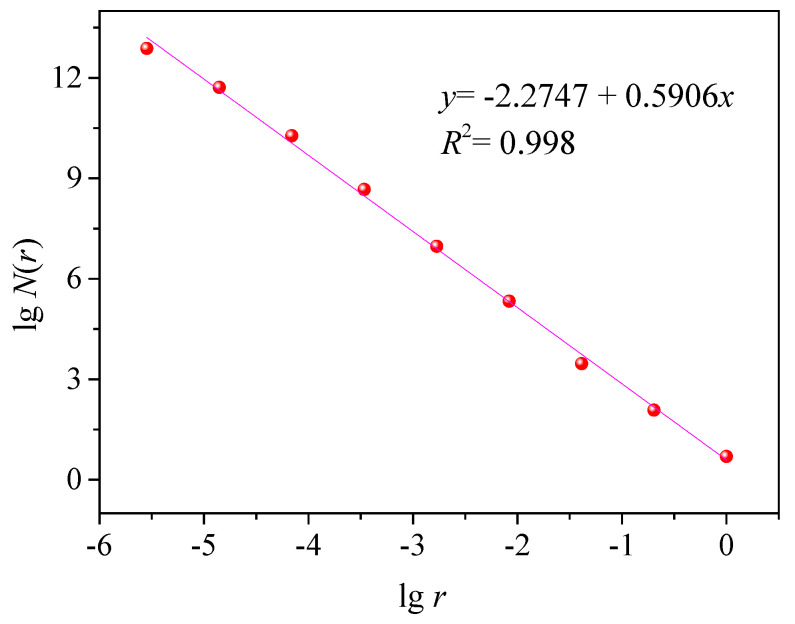
Fitted curve of the fractal dimension obtained via the box-counting method.

N(*r*) ∞ (1/*r*)^D^ (5)

lg*r* and lgN(*r*) serve as the *x*- and *y*-coordinates, respectively. The linear fitting in Figure 11 was conducted using the Origin software. The slope of the fitted trend line, *D*, and correlation coefficient were 2.2747 and 0.998, respectively.

Based on the SEM and the AFM graphs of the No. 5 coating, the coating surface consisted of the UFA particles with an average particle size of 2.35 μm. Approximately 237 nm tiny agglomerates covered the particle surface. The fractal structural particles were formed, the ${\left(\frac{L}{l}\right)}^{D-2}$ was 1.88, and $\theta_{s}\;$ was 118.52°. According to Formula (4), the $\theta_{f}\;$ of the liquid drops on the fractal surface of UFA coating was 153.72°, which was similar to the experimental test value (154.34°). This result indicated that this system conformed to the wettability theory of the fractal structure.

The SEM graph showed that the No. 5 coating had an uneven surface with many pores. Bubbles were observed between the water drops and the coating surface, which formed a solid–gas–liquid three-phase composite interface. The water drops cannot completely soak the coating surface, and this circumstance conformed to the Cassie–Baxter model (Formula (6)) [33,34].

(6) $$\cos\theta =f_{s}\left(\cos\theta_{s}+1\right)-1$$

where *f*_s_ is the ratio of contact area between the water drops and the solids to the total contact area of water drops on the solid surface, that is, the contact area fraction between the water drops and the solids on the composite contact surface.

For a fractal composite rough micro-nanostructural surface, the ${\left(\frac{L}{l}\right)}^{D-2}$ of the fractal structure must be introduced, and the water drop contact angle when water drops unevenly wet the solid surface can then be calculated using Formula (7).

(7) $$\cos\theta =f_{s}{\left(\frac{L}{l}\right)}^{D-2}\left({\cos\theta }_{s}+1\right)-1$$

${\mathrm{where}\;\theta }_{s}$ was 118°, and θ is the apparent contact angle of water drops on the No. 5 coating (154°). The ${\left(\frac{L}{l}\right)}^{D-2}$ of the fractal structure was 1.88, and the $f_{s}$ was 10.05%, which is 89.95% of the area of air cushion.

Bumps (about 2–3 μm) were formed on the surface of the No. 5 coating, and 200–300 nm asperities covered the bumps. Thus, micro-nanocomposite structures with different grades were formed on the coating surface, and the effect of the lotus leaf-like surface structure was generated. A large quantity of air was intercepted between the liquid drops and the coating surface due to the arrangement of this type of composite structures. Liquid drops can only be suspended on the top of composite structures. The liquid drops only had a contact area with the coating of 10% and failed to completely soak the coating. The hydrophobic effect of the coating was improved due to the point contact of water drops caused by the air interception [35].

### 3.4. Application of the Superhydrophobic Composite Coatings onto the Foam Concrete

The superhydrophobic composite coating applied onto the foam concrete included two layers. The UFA-based waterproof and superhydrophobic coatings served as base and surface coatings, respectively. The composite coating was applied onto the surface of the foam concrete through the spray method to exert the waterproofing and heat preservation effects and improve the compressive strengths.

#### 3.4.1. Performance Analysis of the UFA-Based Waterproof Base Coating

**Figure 12:**
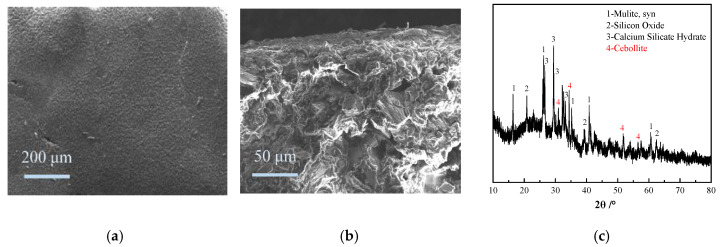
SEM and XRD analyses of the base coating. (**a**) SEM image of coating surface; (**b**) SEM image of coating cross-section; (**c**) XRD pattern of coating.

The UFA-based base coating had a flat surface without cracks (Figure 12a), and its internal structure was compact (Figure 12b). The results of the component analysis of the coating was similar to those of the XRD analysis (Figure 12c). The UFA participating in the reaction generated hydrated silicate with cement, including calcium silicate hydrate (C–S–H gel) and water-containing Ca–Al silicate (cebollite, Ca_5_Al_2_[OH]_4_Si_3_O_12_). Cebollite is a type of fibrous and sheaf-like aggregates and an orthorhombic mineral with Al, Ca, H, O, and Si. A compact matrix was formed on the UFA-containing coating, which provided a powerful support for its strength and durability [36]. In addition, the film formation of the base coating was due to the chemical reaction between the emulsion and the cement hydration product through ionic bonding. The emulsion was evenly mixed with cement and UFA and underwent a crosslinking reaction to generate inorganic–organic composite gel products [RCOO^−^]Ca^2+^[RCOO^−^] under the bridging action of Ca^2+^ [37]. These gel products were deposited and aggregated around the powder particles of the coating system, which filled and blocked the pores in the coating. As a result, reduced pore diameter, refined and compact internal structure, and improved waterproofness were observed in the coating.

The mechanical performance of the UFA-based waterproof base coating was tested. The waterproof base coating was intact without cracks after bending at around mandrel with diameter ≤ 2 mm. The flexibility of the mandrel conformed to the national standard requirements of “Determination of flexibility of films” (GB/T1731-93). The impact resistance of the coating can reach the 50-cm impact height of a heavy hammer. In addition, the coating had no crack or spalling phenomenon and satisfied the national standard requirements of “Determination of impact resistance of films” (GB/T1732-93). The bonding strength of the coating was 1.07 MPa, conforming to the optimal Type III material requirement specified in the national standard “Polymer-modified cement compounds for waterproofing membrane” (GB/T23445-2009).

#### 3.4.2. Influence of the Superhydrophobic Composite Coating on the Foam Concrete

**Figure 13:**
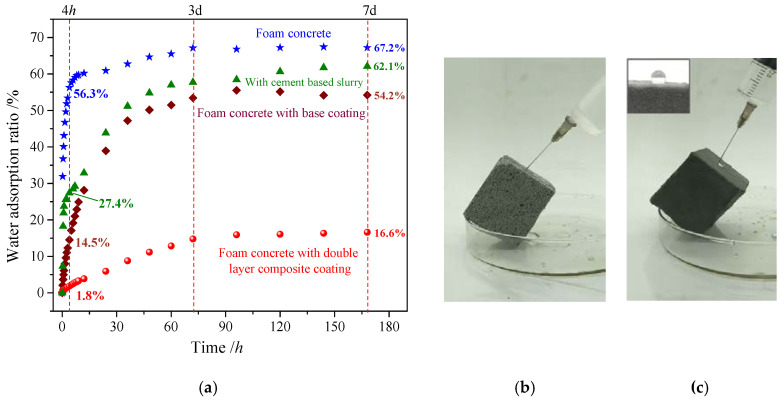
Waterproofness of different blocks: (**a**) Water adsorption ratio of different test blocks; (**b**) Water drop instantly seeps into foam concrete; (**c**) Water drop rolls off the concrete surface with superhydrophobic composite coating.

The density of original foam concrete block is 589.8 kg/cm^3^. The water absorption test of foam concrete test blocks sprayed with different coatings was performed for a period of 7 days [38]. In addition to the original block, test blocks with base coating and/or surface coating, the water absorption rate of the block coated with cement slurry was also test as a comparison. Results are shown in Figure 13a. The water absorption of the foam concrete reached 27.5% after the foam concrete was soaked in water for 5 min. With time, the absorption rate abruptly increased and reached 56.3% after 4 h, and up to 67.2% after 7 days, water absorption saturation was reached. The water absorption ratios of the composite coating test block were 1.8% after 4 h, and 16.6% after 7 days, which were reduced by 97% and 75%, respectively, in comparison with that of the original foam concrete. In addition, the water absorption ratio of the test block coated with cement based slurry was also higher, which is close to that of the test block only coated with base coating. Therefore, the composite coating had considerable waterproofness.

A continuous water drop experiment was also implemented. When the water in the needle tubing was continuously dripped into the foam concrete test block (Figure 13b), the water drops were infiltrated immediately by the test block, and an expanse of imprints was left. When the water drops reached the composite coating test block (Figure 13c), the strings of water drops continuously rolled down, and the surface showed no change. Thus, the composite coating exerted good waterproofness on the concrete test block.

**Figure 14:**
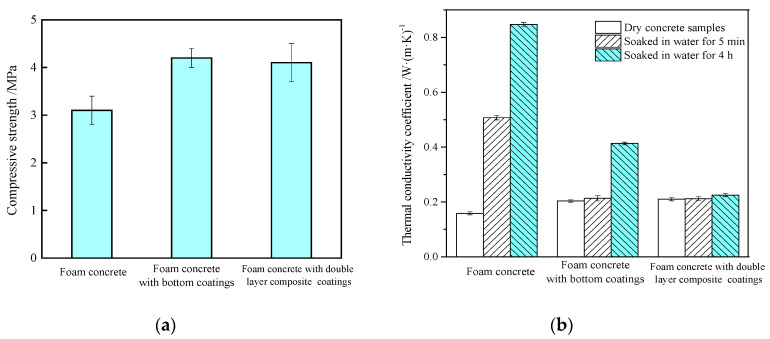
Compressive strengths (**a**) and heat-preserving properties (**b**) of different foam concrete test blocks.

The compressive strengths (Figure 14a) and heat conductivity coefficients (Figure 14b) of different test blocks were determined. The compressive strength of the original foam concrete test block was 3.1 MPa, and the compressive strengths of the bottom and the composite coating test blocks were slightly higher than 4.0 MPa. These results indicated that the coating enhanced the compressive strengths of the test blocks, which became higher than the requirement specified in the international standard for the compressive strength of B05-A2.5.

The heat conductivity coefficient of the foam concrete was 0.158 W· m^−1^·K^−1^. After the test block was soaked in water for 5 min, the heat conductivity coefficient of the original foam concrete test block rapidly increased to 0.507 W m^−1^·K^−1^ and reached 0.847 W m^−1^·K^−1^ after soaking for 4 h. In summary, the heat conductivity coefficient of the original foam concrete test block clearly increased with water, whereas its heat preservation property rapidly degraded. The heat conductivity coefficient of the base coating test block was 0.203 W· m^−1^·K^−1^ and became 0.213 and 0.41 W·m^−1^·K^−1^ after soaking for 5 min and 4 h, respectively.

The heat conductivity coefficient of the composite coating test block was 0.210 W m^−1^·K^−1^ and became 0.212 and 0.225 W·m^−1^·K^−1^ after soaking for 5 min and 4 h, respectively. The heat conductivity slightly changed before and after soaking, which showed that the composite coating in the water environment can guarantee the good waterproofness of the foam concrete test block to provide good heat preservation effect.

### 3.5. Construction Principle of the UFA-Based Superhydrophobic Composite Coating

**Figure 15:**
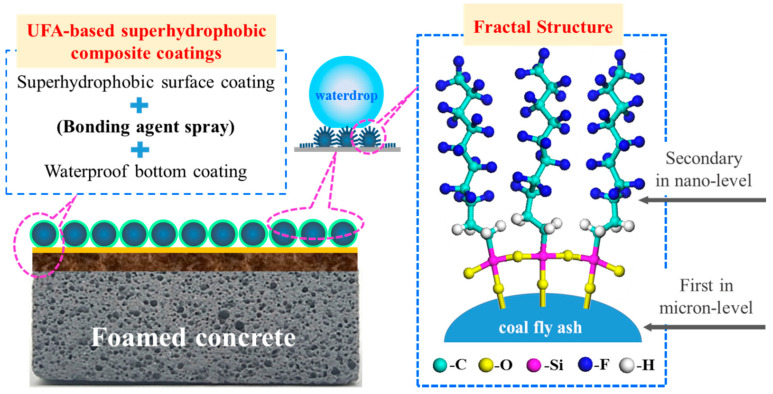
Construction of the UFA-based superhydrophobic composite coating.

The construction of the UFA-based superhydrophobic composite coating is presented in Figure 15. The UFA in the surface coating should undergo fluorinated modification through HFDS. The HFDS dissolved in ethanol was hydrolyzed into Si–OH, and the adjacent Si–OH groups on different silane chains experienced intermolecular dehydration to form intermolecular orderly polycondensates containing the Si–O–Si chain. In addition, the Si–OH formed H bonds with the –OH groups of the UFA surface, followed by the further dehydration and condensation of HFDS to form covalent bonds with UFA. Thus, the hydrophobic well-structured and orderly arranged compact films containing C–F groups were introduced after grafting on the UFA surface, which reduced the surface energy and improved the inorganic–organic interfacial fusion. The theoretical model of chemical bonding is shown in Figure 15 (right). Covalent bonds were formed between the matrix HFDS in the surface coating and the base UFA, which showed that the superhydrophobic surface had good durability.

The application method of the UFA-based superhydrophobic composite coating is shown in Figure 15 (left). The waterproof base coating was sprayed first on the surface of the foam concrete. The early-stage study of our research group has indicated that the interfacial compatibility between the base coating and the OH-containing base material was good and highly cohesive [37]. The bonding agent was sprayed after drying, followed by the superhydrophobic surface coating, thereby forming a sandwich structure “base coating–bonding agent spray–surface coating”. The bonding agent spray played an “intermediate” role in this process with a certain flexibility. The proper bonding agent can be selected on the basis of the practical situation to adjust the bonding strength of the superhydrophobic coating. The surface of the foam concrete sprayed with UFA-based superhydrophobic coating had superhydrophobic performance, and high-efficiency waterproofing and heat preservation effects were fully exerted.

## 4. Conclusions

The UFA-based superhydrophobic composite coating, which included the UFA-based waterproof base and superhydrophobic surface coating, was developed in this study for the effective heat preservation of the foam concrete. Through a series of experiments, the best formula of superhydrophobic surface coating was obtained with 1 mL HFDS, 50mL ethanol, and 5 g UFA. The contact angle of the surface coating was 154.34°, and the sliding angle was 2.41°, indicating superhydrophobicity surface. SEM–EDS and AFM showed that the “lotus leaf-like” micro-nanocomposite structures existed on the coating surface, thereby conforming to the Cassie–Baxter model. These fractal structures endowed different grades of coating surface roughness and about 90% of intercepted air area. The water drops formed the point contact on the coating surface to achieve superhydrophobicity. The superhydrophobic composite coating was applied onto the surface of the foam concrete, and water absorption reduced by 97% (4 h) and 75% (7 d), respectively, in comparison with that of the original foam concrete. The water drops on the foam concrete test block sprayed with composite coating rolled down, successfully certifying the hydrophobicity of the coating. Foam concrete with superhydrophobic composite coating improved the compressive strengths and heat preservation effects.

The UFA-based superhydrophobic composite material was prepared using the spray method. This method was simple and can easily achieve large-scale production without any precise instrument. This method was low cost and exhibited a stable hydrophobic effect, which can provide technical support for heat preservation and energy conservation in buildings. Furthermore, water or ethanol was used to disperse the coating without VOC, which showed its environmental friendliness. UFA was used in the surface and the base coatings, thereby reducing cost and turning waste into “wealth”. The superhydrophobic and antipollution goals can be rapidly achieved through the spray or dip-coating method and can achieve large-scale industrial applications in simple, flexible, and effective ways. The dynamic contact angle test, detailed durability test of this coating, and application of the coating to the surfaces of other soft and hard substrates will be investigated in the follow-up research.

## Figures and Tables

**Table 1:** Chemical composition of UFA.

Composition	SiO_2_	Al_2_O_3_	Fe_2_O_3_	CaO	SO_3_	TiO_2_	K_2_O	MgO	Others
Content (%)	50.1	34.0	4.8	3.7	1.1	1.0	1.0	0.7	3.8

**Table 2:** Surface morphologies and 3D structures of several representative coatings.

Sample Name	SEM Image and Contact Angle	AFM Image and Sq
Blank slide (No. 0 coating)	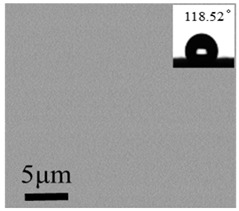	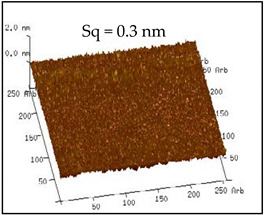
1 g UFA (No. 1 coating)	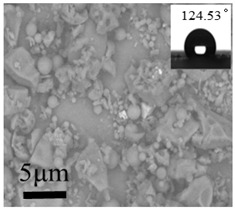	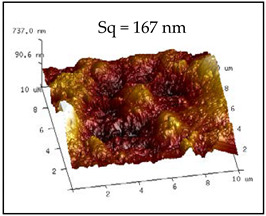
5 g UFA (No. 5 coating)	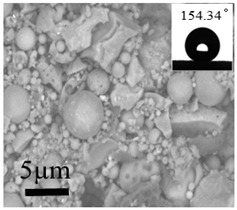	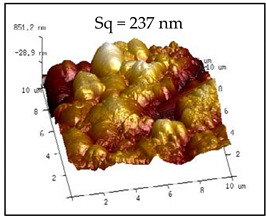
10 g UFA (No. 10 coating)	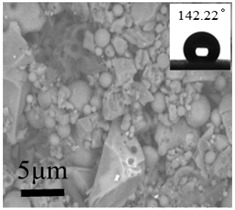	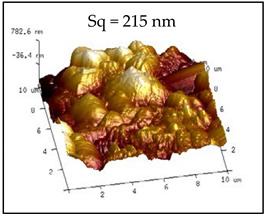

Note: 1 mL HFDS, 50 mL ethanol.

## Supplementary Materials

The following are available online at https://www.mdpi.com/2073-4360/12/10/2187/s1, Video S1: Water droplets falling.
